# Quantification and interpretation of postprandial whole-body protein metabolism using stable isotope methodology: a narrative review

**DOI:** 10.3389/fnut.2024.1391750

**Published:** 2024-05-15

**Authors:** Jorn Trommelen, Luc J. C. van Loon

**Affiliations:** Department of Human Biology, NUTRIM School of Nutrition and Translational Research in Metabolism, Maastricht University Medical Center+, Maastricht, Netherlands

**Keywords:** absorption, anabolism, protein requirements, protein quality, RDA, splanchnic extraction, indicator amino acid oxidation

## Abstract

Stable isotopes are routinely applied to determine the impact of factors such as aging, disease, exercise, and feeding on whole-body protein metabolism. The most common approaches to quantify whole-body protein synthesis, breakdown, and oxidation rates and net protein balance are based on the quantification of plasma amino acid kinetics. In the postabsorptive state, plasma amino acid kinetics can easily be assessed using a constant infusion of one or more stable isotope labeled amino acid tracers. In the postprandial state, there is an exogenous, dietary protein-derived amino acid flux that needs to be accounted for. To accurately quantify both endogenous as well as exogenous (protein-derived) amino acid release in the circulation, the continuous tracer infusion method should be accompanied by the ingestion of intrinsically labeled protein. However, the production of labeled protein is too expensive and labor intensive for use in more routine research studies. Alternative approaches have either assumed that 100% of exogenous amino acids are released in the circulation or applied an estimated percentage based on protein digestibility. However, such estimations can introduce large artifacts in the assessment of whole-body protein metabolism. The preferred estimation approach is based on the extrapolation of intrinsically labeled protein-derived plasma bioavailability data obtained in a similar experimental design setting. Here, we provide reference data on exogenous plasma amino acid release that can be applied to allow a more accurate routine assessment of postprandial protein metabolism. More work in this area is needed to provide a more extensive reference data set.

## 1 Introduction

All living tissues are in a constant state of protein turnover, regulated by the balance between protein synthesis and breakdown rates. This turnover provides tissues with plasticity, e.g., by replacing damaged protein or protein remodeling in response to stress. Furthermore, tissue can hypertrophy or atrophy, based on a prolonged net positive or negative protein balance, respectively. In a fasted state, protein balance is negative, resulting in a net loss of protein mass (catabolism). An influx of exogenous amino acids is required for protein balance to become positive (anabolism) and offset fasted losses. Dietary protein intake is essential to maintain lean body mass, with the current recommended daily allowance (RDA) estimated at 0.8 g·kg^−1^·day^−1^ (1). However, it is generally believed that the RDA is insufficient to attenuate lean body mass loss during conditions such as energy restriction or aging (2, 3). Moreover, protein intakes exceeding the recommended daily allowance may further stimulate anabolism and elicit benefits such as improving the adaptive response to exercise, improving immune function, and accelerating wound healing (4–8). Therefore, there is much interest in the determination of the optimal dietary protein intake to maximize health and function and how this is modulated by factors such as protein quality, protein timing, and/or protein distribution. However, there is much debate on the methodology to accurately assess protein requirements and the impact of protein quality on post-prandial protein handling. Despite known limitations, recommendations for protein requirements and quality are currently primarily based on nitrogen balance, the Indicator Amino Acid Oxidation (IAAO) method, and Digestible Indispensable Amino Acid Score (DIAAS) (1, 9). Theoretically, the accurate assessment of whole-body protein metabolism would provide an ideal method to not only assess protein requirements and protein quality, but also provide insight in the underling metabolic rates (protein synthesis, breakdown, oxidation, and net balance). Whole-body protein metabolism can be quantified using stable isotope methodology (10, 11). By applying a constant amino acid tracer infusion and taking frequent blood samples, the assessment of postabsorptive whole-body protein metabolism is relatively simple. In contrast, the assessment of postprandial whole-body protein metabolism is more challenging when exogenous protein-derived plasma amino acid bioavailability (hereafter referred to as “exogenous plasma amino acid bioavailability”) needs to be taken into account. Here we discuss (1) the plasma amino acid kinetics model to determine whole-body protein metabolism in the postabsorptive and postprandial state, (2) the impact of exogenous plasma amino acid bioavailability in the amino acid kinetics model, and (3) the various approaches available to determine and/or estimate exogenous plasma amino acid bioavailability and subsequently postprandial protein metabolism.

## 2 The plasma amino acid kinetics model

**Figure 1:**
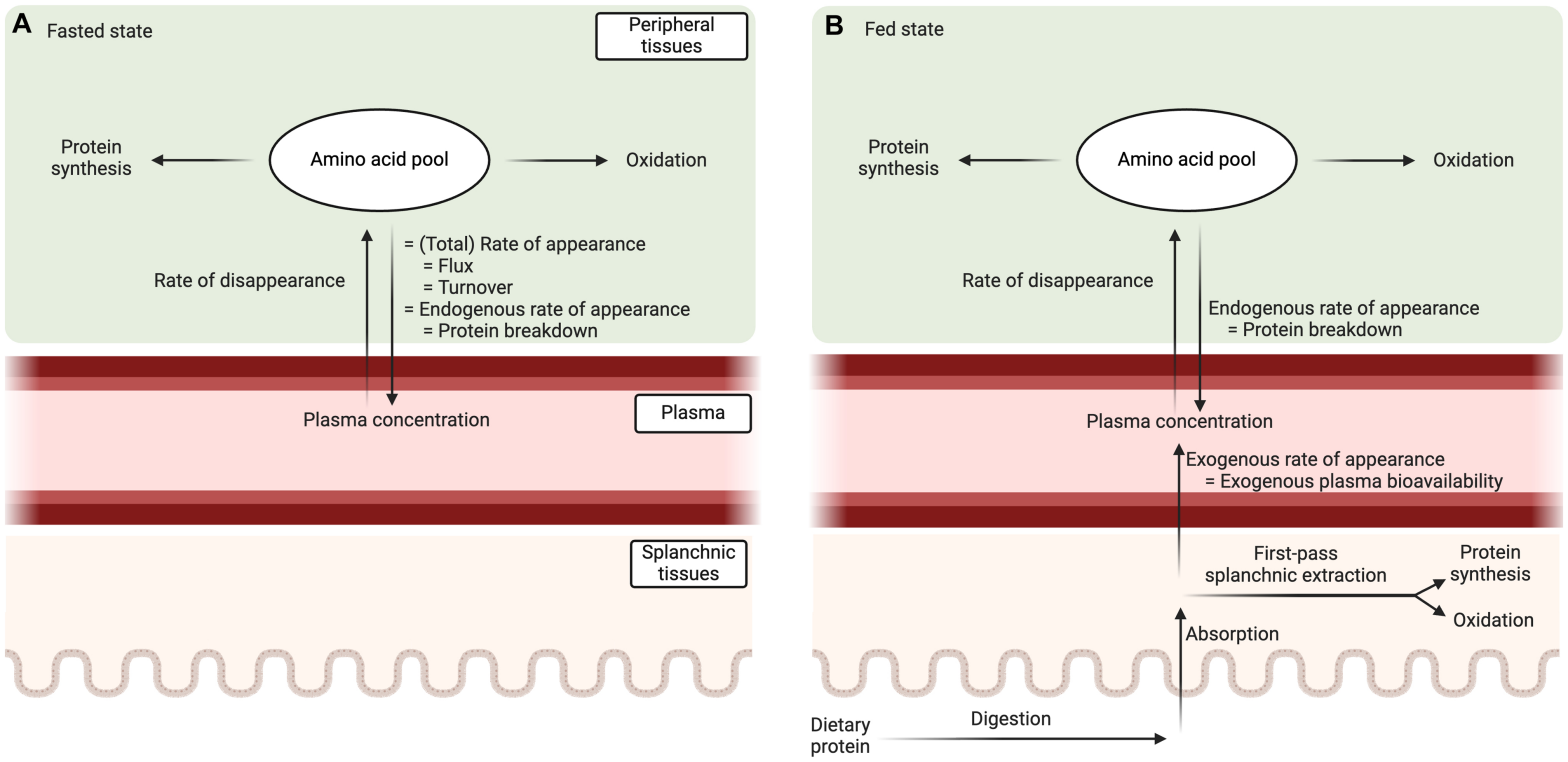
Schematic representation of the plasma amino acid kinetics model in fasted **(A)** and fed state **(B)**. In the fed state, the total amino acid rate of appearance into the circulation consists out of an endogenous (tissue protein breakdown) and exogenous (dietary protein-derived plasma amino acid availability) component. Exogenous plasma amino acid bioavailability needs to be assessed or estimated to allow the calculation of whole-body protein breakdown rates.

Whole-body protein metabolism can be assessed based on plasma amino acid kinetics, i.e., the rates at which amino acids are released into and taken up from the circulation (10, 11). In the fasted state (Figure 1A), amino acid release into the circulation originates solely from tissue protein breakdown (endogenous protein-derived plasma amino acid rate of appearance). Thus, the total amino acid rate of appearance, the endogenous rate of appearance, and whole-body protein breakdown rate are all equal in the fasted steady state. The rate at which amino acids disappear from the circulation represents the rate of amino acid uptake into tissues. Amino acids taken up by tissues are assumed to be either incorporated into proteins (protein synthesis) or oxidized. Amino acid oxidation can be measured by the irreversible hydroxylation of phenylalanine to tyrosine (12) or by the production of ^13^CO_2_ in expired air (13). Subsequently, protein synthesis rate can be calculated by subtracting the rate of amino acid oxidation from the rate of disappearance. Finally, protein balance can be assessed by subtracting protein breakdown from protein synthesis. The calculations to assess plasma amino acid kinetics and whole-body protein metabolism in a fasted (and fed) state have been described in detail before (11).

In the fed state (Figure 1B), the assessment of whole-body protein kinetics is more challenging because amino acids not only appear into the circulation from protein breakdown (endogenous protein-derived plasma amino acid appearance), but also from the ingested protein (exogenous plasma amino acid appearance). This exogenous plasma amino acid rate of appearance needs to be quantified and accounted for (subtracted from the total plasma amino acid appearance rate) to calculate postprandial protein breakdown rates and, consequently, net protein balance. The exogenous plasma amino acid rate of appearance cannot be directly assessed with the amino acid stable isotope approach used to assess the total plasma amino acid kinetics. Therefore, the tracer methodology needs to be extended to directly assess the exogenous plasma amino acid bioavailability or alternatively the exogenous plasma amino acid bioavailability needs to be estimated.

## 3 Exogenous plasma amino acid bioavailability

**Figure 2:**
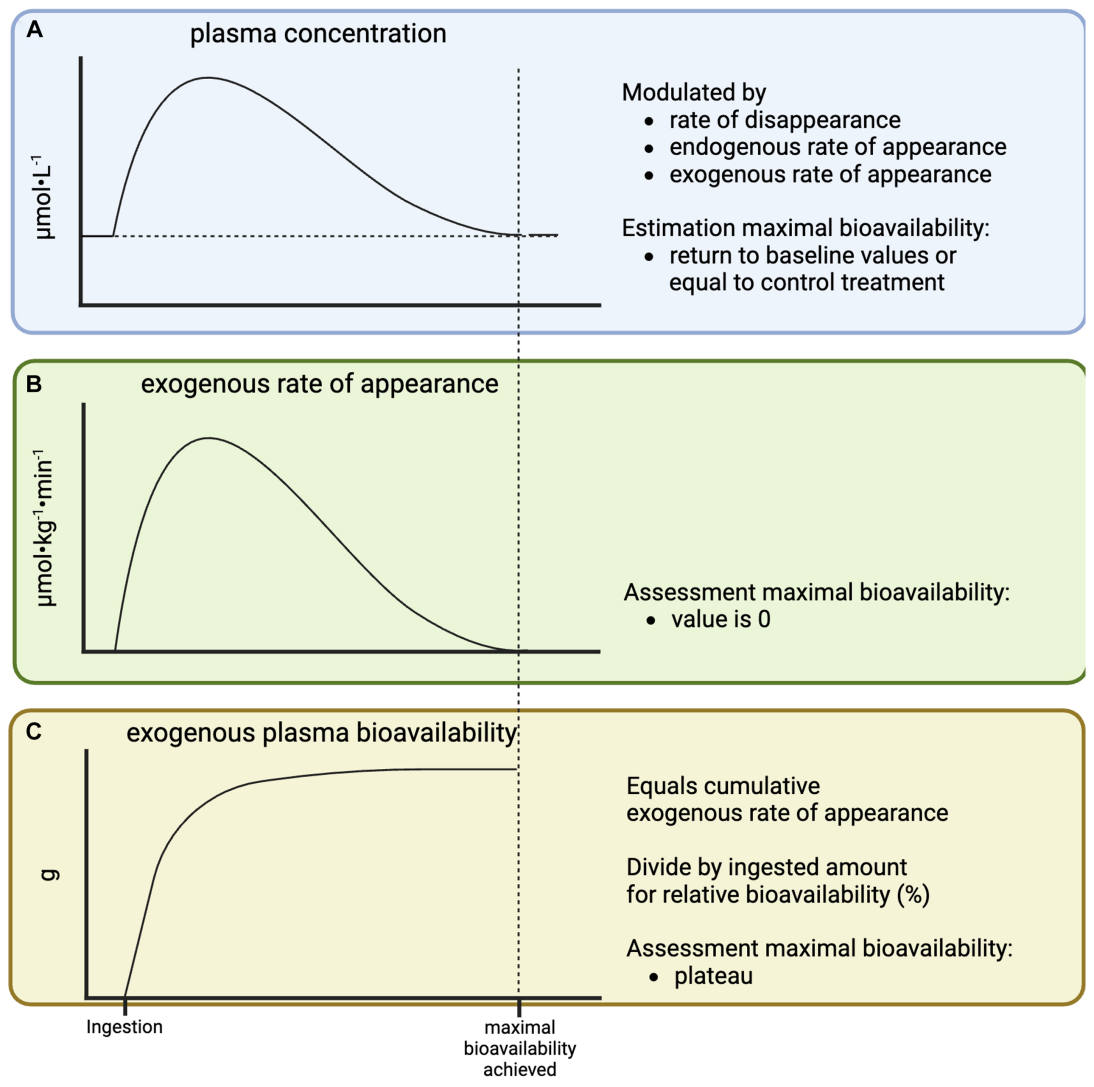
Schematic representation of plasma amino acid concentration **(A)**, exogenous rate of plasma amino acid appearance **(B)**, and exogenous plasma amino acid bioavailability **(C)** in response to the ingestion of a single bolus of protein.

Plasma amino acid concentrations are often used as a proxy for exogenous plasma amino acid bioavailability (Figure 2A), as it does not require the application of (more) amino acid tracers. However, plasma amino acid concentrations are not only impacted by exogenous plasma amino acid release, but also by endogenous amino acid release into the circulation (tissue protein breakdown) and the rate at which amino acids are taken up by tissues. Therefore, plasma amino acid concentrations cannot quantify exogenous plasma amino acid bioavailability. However, changes in plasma amino acid concentrations over time can provide some insight in the time course of exogenous plasma amino acid release, which is important for the interpretation of postprandial protein metabolism as will be discussed later. Following the ingestion of protein, plasma amino acid concentrations will rise and subsequently return to baseline. A complete return to baseline concentrations suggests that the ingested protein has been fully digested, absorbed, and released into the circulation (maximal exogenous plasma amino acid bioavailability has been reached). However, the experimental baseline sample may not always be representative of basal conditions. For example, many studies investigate the impact of protein ingestion directly following exercise when plasma amino acid concentrations are elevated due to exercise-induced catabolism (12). Therefore, the time point at which plasma amino acid concentrations in a postexercise feeding treatment do no longer differ from a placebo treatment would give a better indication of when maximal exogenous plasma amino acid bioavailability has been reached. Ideally, exogenous plasma amino acid bioavailability is assessed directly using tracer methodology. Following protein ingestion, exogenous plasma amino acid rate of appearance becomes positive and will eventually return to its baseline of zero, indicating maximal exogenous plasma amino acid bioavailability has been reached (Figure 2B). The area under the curve of the exogenous rate of appearance represents exogenous plasma amino acid bioavailability in absolute amounts (g). This can be divided by the ingested amount of protein to express it in a relative amount (percentage of the ingested protein). A plateau in the cumulative timeline of exogenous plasma amino acid bioavailability represents maximal exogenous protein/AA bioavailability (Figure 2C).

## 4 Impact of protein bioavailability on the assessment of postprandial protein metabolism

**Figure 3:**
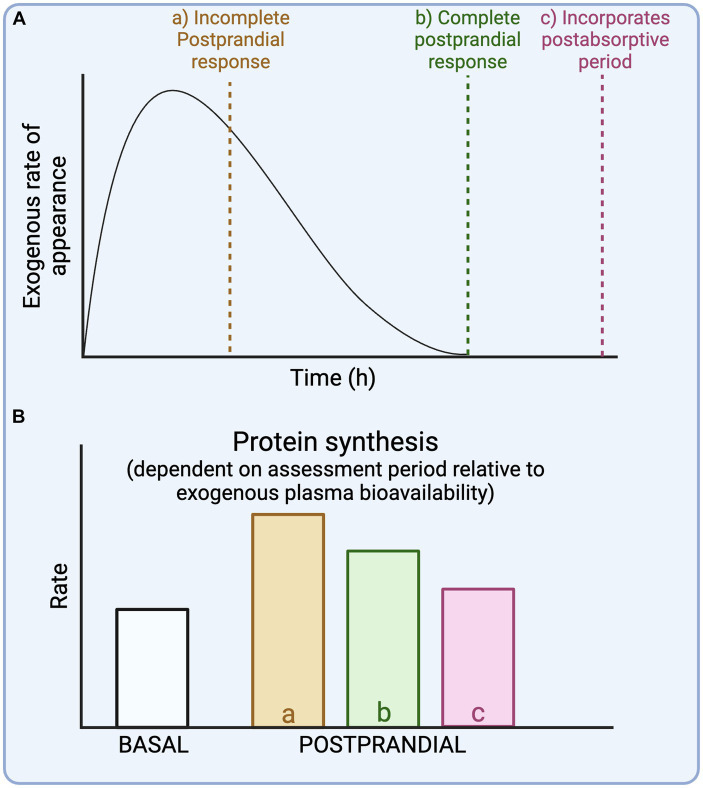
Schematic representation of different experimental durations relative to exogenous plasma amino acid bioavailability **(A)** and their impact on postprandial whole-body protein synthesis rates **(B)**.

Insight in the timeline of exogenous plasma amino acid bioavailability is required to properly evaluate whole-body protein metabolic rates in the experimental context. As meals are typically consumed as a (single) bolus, this introduces a non-steady state and results in a time-dependent variation in whole-body protein metabolic rates. To characterize a more complete postprandial response to a meal, the assessment period should at least match the time required to achieve maximal exogenous plasma amino acid bioavailability (Figure 3A, dotted line b). But when the assessment period is longer than the time point at which maximal exogenous protein derived plasma amino acid bioavailability is reached, this introduces a postabsorptive period within the assessment (Figure 3A, dotted line c). Consequently, this will lower the average protein synthesis rates during the “assumed postprandial” assessment period. The impact of an experimental period that is too short to reach maximal exogenous plasma amino acid bioavailability (Figure 3A, dotted line a) will depend on the pattern of protein-derived amino acid release into the circulation (fast vs. slow). For example, most protein-derived amino acids are released in the initial hours following the ingestion of a more rapidly digestible protein (14, 15). When the assessment period is short and matching this peak amino acid availability, this would overestimate average whole-body protein synthesis rates during the complete postprandial period. Conversely, the exogenous plasma amino acid rate of appearance following the ingestion of a more slowly digestible protein or a large whole-foods mixed meal may not peak until several hours into the post-prandial period (14–16). When the assessment period would end before the peak exogenous plasma amino acid availability, it may result in a gross underestimation of the average whole-body protein synthesis rates during the complete postprandial period and total whole-body protein synthetic response to the meal. In support, we have recently demonstrated that the ingestion of a large amount of protein (100 g milk protein) results in a much larger and more prolonged (>12 h) protein synthetic response than was previously assumed based on shorter experiments (12). Thus, the expected time course of exogenous plasma amino acid bioavailability is a crucial consideration in study design and the interpretation of data (Figure 3B).

While the time course of exogenous plasma amino acid bioavailability only impacts the interpretation of whole-body protein synthesis and amino acid oxidation rates, exogenous plasma amino acid bioavailability needs to be quantified to for the calculation of whole-body protein breakdown rates (11). Specifically, whole-body protein breakdown is calculated by:


(1) $$\mathrm{Protein}\;\mathrm{breakdown}=\mathrm{Tota}l_{Ra}-\mathrm{Ex}o_{Ra}$$

**Figure 4:**
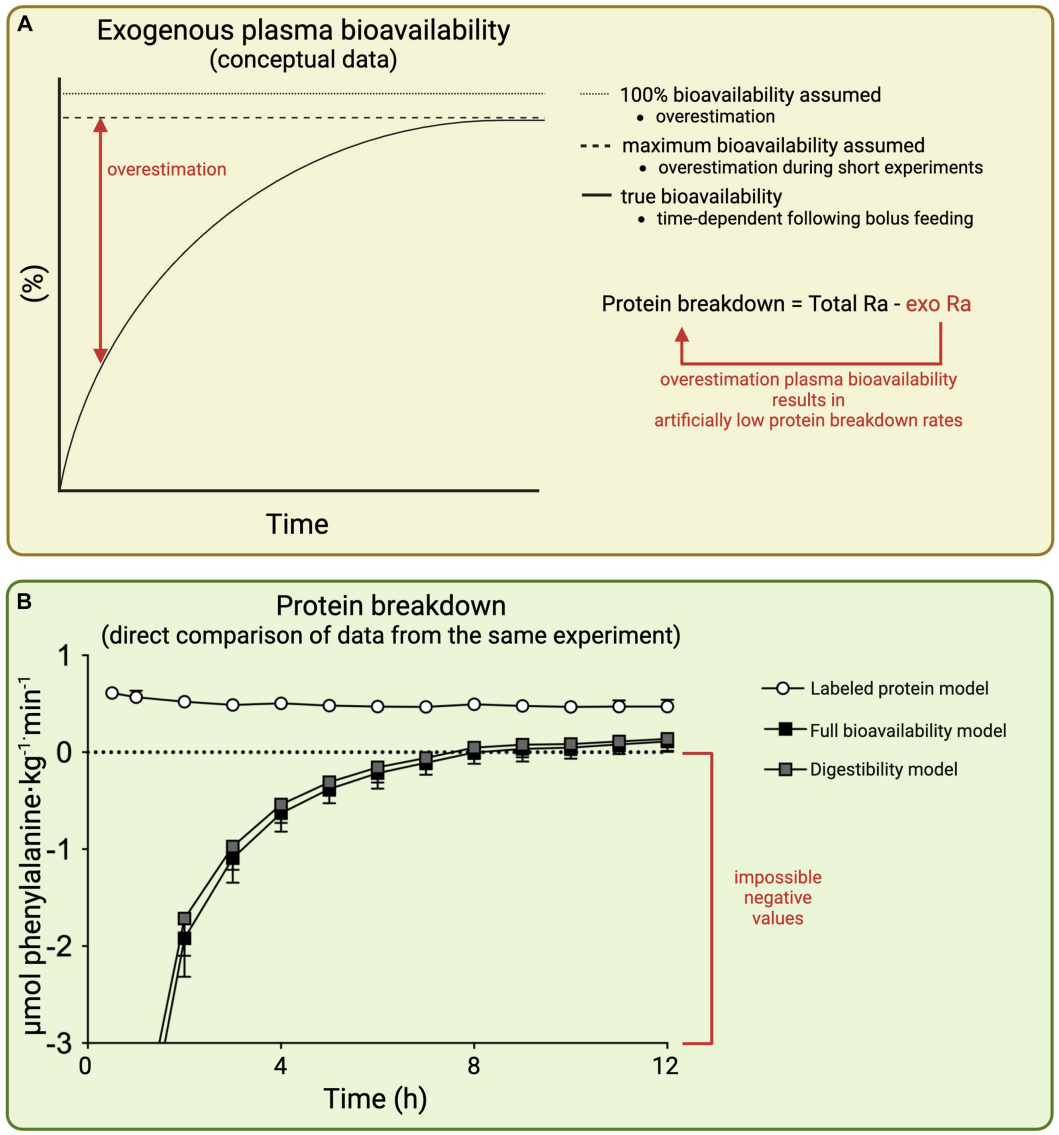
Schematic representation of the impact of inaccurate estimation of exogenous plasma amino acid bioavailability on whole-body protein breakdown rates **(A)** and whole-body protein breakdown rates as calculated based on different plasma amino acid kinetic models following the ingestion of 100 g protein; all three models calculated from the same raw data from Trommelen et al. (12) (9) **(B)**. Total Ra: total rate of both endogenous plus exogenous amino acids appearing into the circulation. Exo Ra, rate of exogenous amino acids appearing into the circulation.

Total_Ra_ and Exo_Ra_ represents the total and exogenous plasma amino acid rate of appearance, respectively. Assessment of the exogenous amino acid rate of appearance allows the time course of whole-body protein breakdown rates to be determined. When only a single estimated value for exogenous plasma amino acid bioavailability is available, only an average whole-body protein breakdown rate during the entire assessment period can be calculated. This does not impact the validity, but time-course data can provide additional valuable insights, such as, whether the effects are short-lived, increase over time, or correspond with other variables such as insulin levels. As can be deduced from the formula, any inaccuracy in the assessment or estimation of plasma bioavailability directly translates in inaccurate whole-body protein breakdown rates (Figure 4A). However, there are various plasma amino acid kinetic models routinely applied that differ greatly in their estimations of exogenous plasma amino acid bioavailability.

## 5 Plasma amino acid kinetics models to assess postprandial whole-body protein metabolism

### 5.1 100% bioavailability model

Initial work on whole-body protein metabolism developed a simplified plasma amino acid kinetics model that did not account for the bioavailability of amino acid released from the ingested protein (17):


(2) Q=PS+OX=PB+ING

*Q* represents whole-body flux/turnover (or total rate of amino acid appearance as used in contemporary models). PS represents protein synthesis, OX represents oxidation (catabolism), PB represents protein breakdown, and ING represents protein ingestion. Note that despite older terminology for the elements, formula 2 can be rearranged to construct formula 1, with the exception that formula 2 does not account for the plasma amino acid bioavailability of the ingested protein. Therefore, all ingested protein is assumed to appear into the circulation, which generally is a substantial overestimation and results in incorrect assessment of whole-body protein breakdown. To illustrate, we applied the 100% bioavailability model to our data set of our recent work in which 100 g of protein was ingested (12) (Figure 4B). This allows a direct comparison of plasma amino acids kinetics and whole-body protein metabolism as assessed/estimated by various models based on the same raw data. This resulted in negative values for protein breakdown rates in the 100% bioavailability model, which is physiologically impossible. In contrast, protein breakdown rates were only reduced by ∼5%, assessed using the gold standard labeled protein method (methodology discussed in section 5.3). In general, protein ingestion has only a modest impact on protein breakdown, with reductions between 5 and 25% in whole-body protein breakdown rates observed following protein ingestion as assessed with the labeled protein method (18–20). The magnitude of error in the full bioavailability model is largest during short experimental methods where the overestimation of exogenous plasma amino acid bioavailability is greatest. As the model ignores the true exogenous plasma amino acid bioavailability, it is fundamentally flawed. Therefore, the full bioavailability model should be considered outdated, as there are alternative approaches that do not require additional measurements but estimate exogenous plasma amino acid bioavailability to improve accuracy of the model.

It should be noted that the model with assumption of full exogenous amino acid bioavailability is still applied with some frequency, most commonly when applying the Indicator Amino Acid Oxidation (IAAO) method (11, 21). While the full exogenous plasma amino acid bioavailability model was designed for study designs in which amino acid tracer infusions were applied, the IAAO method typically applies only the ingestion of an amino acid tracer. However, not all ingested amino acids (tracers) appear into the circulation as the exogenous amino acid bioavailability in the circulation never reaches 100% [maximal exogenous plasma amino acid bioavailability is ~80% (18)]. Therefore, this approach has the inherent limitations of the 100% bioavailability method, but also violates the model assumption of 100% plasma bioavailability of the tracer (as the model was developed for intravenous tracer infusion). In support, plasma amino acid kinetics differ substantially in the IAAO model when comparing an intravenous vs. oral tracer approach (22). Nevertheless, the intake level that results in a breakpoint in indicator amino acid oxidation (assumed to represent the protein or essential amino acid requirement) is consistent between the intravenous and oral tracer method. It has been suggested that the oral amino acid tracer approach can still be applied to evaluate changes in (oral tracer-derived) whole-body protein metabolism (21). However, this approach has not been validated against gold-standard dual tracer feeding-infusion methods and, therefore, should be considered exploratory. In conclusion, IAAO-derived plasma amino acid kinetics rates are likely not accurate for either the oral or infusion method and should not be reported as secondary outcomes. Both models give consistent estimates for the indicator amino acid oxidation breakpoint, which suggests that they may be valid for the assessment protein and essential amino acid requirements.

### 5.2 The digestibility model estimates exogenous plasma amino acid bioavailability

The 100% bioavailability model can be improved by correcting the amount of ingested protein for estimated exogenous plasma amino acid bioavailability. Exogenous plasma amino acid bioavailability (Formula 3) represents the ingested protein-derived amino acids that are absorbed in the gut, subsequently escape first pass splanchnic extraction, and are released into the systemic circulation (23, 24):


(3) $$\mathrm{BI}O_{\mathrm{estimated}}=ING*\mathrm{digestibility}*SPE_{\mathrm{estimated}}$$

BIO_estimated_ represents the estimated exogenous plasma amino acid bioavailability, digestibility represents the true ileal digestibility of the ingested protein, and SPE_estimated_ represents the estimated first pass splanchnic extraction. By dividing BIO_estimated_ by the duration of the assessment period, it is converted to the (estimated) average exogenous rate of plasma amino acid appearance. The latter can be used in formula 1 to calculate the average protein breakdown rate over the assessment period. While true ileal protein digestibility has long been challenging to assess in humans due to the requirement of invasive techniques, there are data from animal (especially pig) models that seem to correspond well with data derived from human *in vivo* models (25). Moreover, the development of the minimally invasive dual tracer digestibility techniques has allowed more human data to be collected in recent years (26, 27). As digestibility represents the exogenous protein-derived amino acids that may be absorbed in the gut, an additional correction for first-pass splanchnic extraction needs to be applied to estimate subsequent exogenous plasma amino acid bioavailability. This first-pass splanchnic extraction can be estimated based on the postprandial increase in whole-body amino acid oxidation (postprandial – postabsorptive rates) (28, 29).

An advantage of the digestibility method is that there are considerable amounts of data available for the digestibility of most proteins. Therefore, the digestibility approach can be applied in most experiments. The main drawback is that the method relies on multiple assumptions and extrapolations that have the potential to introduce errors. Digestibility data are typically obtained during steady state conditions which do not reflect the bolus feeding approach used in stable isotope studies to reflect the response to ingesting a normal meal. The digestibility obtained during steady state conditions represents the maximal protein digestibility of the protein source when given sufficient time. Therefore, maximal digestibility data should not be extrapolated to a bolus feeding study of relatively short duration with insufficient time to allow maximal digestibility or maximal exogenous plasma amino acid bioavailability to be reached. For example, milk protein may have a 95% digestibility given sufficient time (25), but clearly not all protein is digested, absorbed, and released into the circulation within the first hour after the ingestion of a large milk protein bolus (12). Thus, extrapolating the maximal digestibility data to short experimental duration results in overestimation of exogenous plasma amino acid bioavailability and consequently results in artificially low protein breakdown rates (Figure 4). Other limitations of the model are the assumptions that are made regarding first-pass splanchnic extraction. It needs to be assumed that splanchnic tissues are in net balance (28), although there is some indication that net balance may be negative in the postprandial state (30). In addition, the postprandial increase in whole-body amino acid oxidation is assumed to reflect first-pass oxidation. However, oxidation is assessed by the application of a continuous amino acid tracer infusion directly into the circulation. Therefore, any tracer-derived oxidation in the model cannot be the result of first-pass splanchnic extraction. While the digestibility model has substantial limitations, the method is conceptually superior to approaches that do not account for exogenous plasma amino acid bioavailability.

### 5.3 Intrinsically labeled protein to assess exogenous plasma amino acid bioavailability

Exogenous plasma amino acid bioavailability can be assessed by combining the application of an amino acid tracer constant infusion with the ingestion of a different stable isotope of the same amino acid (e.g., L-[^2^H_5_]-phenylalanine and L-[1-^13^C]-phenylalanine, respectively). The ingested amino acid tracer should reflect the properties of the amino acids it traces which requires them to be in the same matrix. The ingestion of a free amino acid tracer can be applied to assess the exogenous amino acid bioavailability following the ingestion of a free amino acid mixture, but not following the ingestion of a protein source. In a real-life setting, exogenous amino acids are typically consumed in the form of dietary proteins. Therefore, the assessment of postprandial whole-body protein metabolism following the ingestion of intact dietary proteins is of particular relevance. This requires the dietary protein to be intrinsically labeled, i.e., the amino acid tracer should be incorporated into the protein matrix. The intrinsic labeling of dietary protein can be achieved in multiple ways, such as feeding or infusing amino acid tracers to, for example, insects (31), chickens (32), or cows (33). The intrinsic labeling of plant proteins is also possible and has been applied to assess protein digestibility (27), but not yet for exogenous plasma amino acid bioavailability. Exogenous plasma bioavailability is calculated as follows:


(4) $$\mathrm{Ex}o_{Ra}=\mathrm{tota}l_{Ra}*\frac{E_{\mathrm{plasma}}}{E_{\mathrm{pro}}}$$

(5) $$\begin{array}{l} \mathrm{Exogenous}\;\mathrm{plasma}\;\mathrm{AA}\;\mathrm{bioavailbilit}y_{\left(t\right)}=\mathrm{area}\;\mathrm{under}\;\mathrm{the}\;\\ \mathrm{curve}\;\mathrm{of}\;\mathrm{Ex}o_{Ra} \end{array}$$

Exogenous plasma AA bioavailability_(t)_ represents the cumulative amount of dietary protein-derived amino acids that have been released in the circulation at a specific time point (Formula 5). *E*_plasma_ represents the enrichment of the labeled protein-derived tracer in the circulation. *E*_pro_ represents the enrichment of the labeled protein before ingestion. The combination of an amino acid tracer infusion with the ingestion of intrinsically labeled protein is the preferred method to quantify postprandial protein metabolism, as it is the only method to directly quantify exogenous plasma amino acid bioavailability. The drawback of this method is that the production of intrinsically labeled protein is expensive and labor intensive to apply. Therefore, there is a need for alternative approaches that can provide a more routine evaluation of postprandial whole-body protein metabolism.

**Figure 5:**
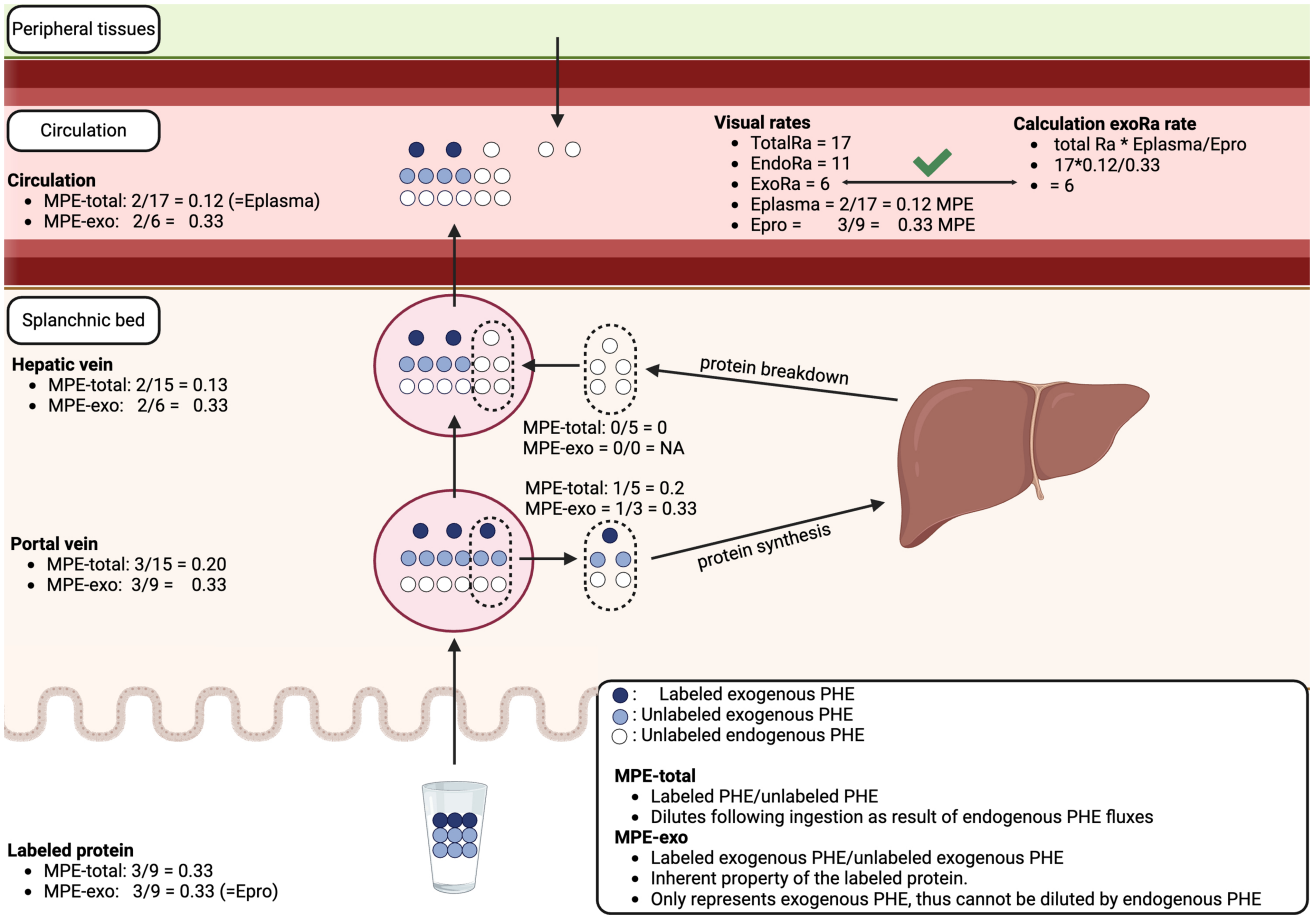
Schematic overview of exogenous protein-derived amino acid release into the circulation as assessed with intrinsically labeled protein model. The enrichment of the exogenous amino acid is an inherent property of the labeled protein and cannot be impacted by endogenous fluxes. The validity of the calculation of exogenous rate of plasma amino acid appearance (ExoRa) can be visually confirmed. The model can be extended to include additional amino acid kinetics or other physiological changes while remaining valid. MPE, Mole percent excess; PHE, Phenylalanine; TotalRa, Total rate of plasma amino acid appearance; EndoRa, Endogenous rate of plasma amino acid appearance; Eplasma, Enrichment of the exogenous protein-derived amino acid tracer in the circulation; Epro, Enrichment of the exogenous amino acid tracer in the dietary protein.

It should be noted that the accuracy of the intrinsically labeled protein to assess exogenous plasma amino acid bioavailability has been questioned (28, 34). It was suggested that the enrichment of the labeled protein-derived tracer gets diluted across the splanchnic bed, which would result in an underestimation of exogenous plasma amino acid bioavailability. While the enrichment of the labeled-protein derived tracer gets diluted following ingestion, this has no impact on the assessment of exogenous plasma amino acid bioavailability or *E*_pro_ as used in formula 4. *E*_pro_ represents the enrichment of the labeled protein before ingestion, which also can be defined as the enrichment of the exogenous amino acids or the tracee. By definition, the enrichments of the exogenous tracee are not diluted by any endogenous flux. The enrichment of the exogenous tracee is used in the formula to calculate the plasma appearance rate back from tracer to tracee. For example, an E_pro_ of 50% MPE indicates that for every tracer appearing in the circulation (calculated by total_Ra_ * E_plasma_), and equal amount of exogenous tracee appears into the circulation. Therefore, the exogenous rate of appearance is two times (equals dividing by 50% MPE) the exogenous rate of appearance of the tracer. Figure 5 demonstrates that E_pro_ is not diluted throughout the splanchnic bed and that calculation of the exogenous rate of appearance is accurate using the intrinsically labeled protein model. The model can be challenged by modifying variables like the protein intake dose, the labeled protein enrichment, any of the metabolic rates, and/or incorporating additional factors such as digestibility, additional endogenous rate of appearance, additional rates of disappearance, or a net splanchnic extraction/release, but the model remains valid under all these challenges. Therefore, the intrinsically labeled protein method can be used to accurately quantify exogenous plasma amino acid bioavailability.

### 5.4 Estimation of exogenous plasma amino acid bioavailability based on published literature

When exogenous plasma amino acid bioavailability cannot be directly assessed by using intrinsically labeled protein, the most accurate estimation would be based on reference data from such dual tracer isotope-feeding models that have been performed under similar experimental context. This is conceptually the same approach as the digestibility method but requires less assumptions. The main limitation of this method is that there are not that many reference data available. Only for bovine milk protein, there are substantial data obtained during various experimental conditions (e.g., different doses, protein fractions, nutrient co-ingestion, age, different assessment periods, exercise, and sleep). There are few data on exogenous plasma amino acid bioavailability following the ingestion of most other protein sources, and plant-based protein sources in particular. This is further complicated by the fact that exogenous plasma amino acid bioavailability data are not always (completely) reported. For example, even when the labeled protein approach is applied, exogenous rates of plasma amino acid appearance are not always calculated and/or reported. Moreover, studies have only recently started to report data on the cumulative exogenous plasma amino acid bioavailability. To address this, we have compiled our previous data (12, 18–20, 31, 35–51) and provide data on cumulative exogenous plasma amino acid bioavailability expressed as a percentage of the ingested protein in Table 1. These data allow estimation of the average exogenous rate of plasma amino acid appearance which can be used in formula 1, similar as discussed for the digestibility approach (example provided below). There is a need to establish a database on exogenous plasma amino acid bioavailability of the most common protein sources in *in vivo* in various populations and experimental conditions.

**Table 1:** Overview of exogenous plasma amino acid bioavailability data at different time points following bolus protein during various experimental settings and designs.

Study	Type	Dose (g)	Age (years)	Exercise	Other	Cumulative plasma bioavailability (%)					
						3 h	4 h	5 h	6 h	8 h	12 h
(18)	AA	30	23 ± 3			63	70	73	76		
	Milk					36	46	53	59		
(45)	Whey	10	73 ± 5			53	56				
		20				53	58				
		35				48	54				
(39)	Whey	25	62 ± 5		low PRO	53	56	58			
					high PRO	49	52	54			
(49)	Whey	24	22 ± 3		immob	54	58				
(43)	Casein	35	23 ± 3			24	30	35	40		
			64 ± 4			24	30	36	40		
(46)	Casein	20	21 ± 2	-		33	41	46	49		
			21 ± 3	REX		29	37	43	47		
			75 ± 4	-		36	42	46	50		
			73 ± 3	REX		32	39	43	47		
(38)	Casein	20	21 ± 3		-	47	56	61			
			20 ± 2		CHO	41	55	64			
			74 ± 3		-						
			76 ± 4		CHO	38	45	51			
						33	46	54			
(40)	Casein	20	65 ± 7		-	36	43	47			
					FAT	34	42	48			
(50)	Casein	25	71 ± 6		-	30	36	41			
					serum	27	36	42			
(41)	Casein	20	23 ± 3		-	34	42	48			
			21 ± 2		insulin	36	49	61			
			68 ± 4		-						
					insulin	27	34	40			
			68 ± 2		-	26	34	40			
(47, 48)	Casein	30	23 ± 4	-	sleep	26	34	40	46	54	
				REX	sleep	27	35	41	47	54	
				REX	sleep+leu	28	37	44	51	58	
(42, 44)	Casein	20	72 ± 5	REX	sleep	37	46	52	57	61	
		20			sleep+leu	28	37	45	51	58	
		40			sleep	20	28	35	41	51	
		40			sleep	23	31	38	45	52	
(51)	Casein	20	24 ± 3		-	40	49				
					immob	42	47				
(19)	Milk	15	27 ± 4	END	CHO	62	68	71	74		
		30				64	69	72	74		
		45				55	63	67	69		
(12)	Milk	25	26 ± 6	REX		42	51	56	58	62	66
		100				21	26	31	36	44	53
(20)	Milk	15	66 ± 6	REX	-	59	67	72	75		
		30									
					-	41	51	58	63		
		45									
					-	41	49	55	59		
		15									
					leu	52	61	67	70		
(36)	Milk	20	21 ± 2	REX	CWI + CHO	62	68	71			
(37)	Milk	20	23 ± 3	REX	HWI ± CHO	66	71	73			
(35)	Milk	30	22 ± 3	REX		51	60	65			
	Beef*					39	40	58			
(31)	Worm*Milk	30	23 ± 3	REX		58	68	73			
						58	70	77			

Age: mean ± SD. ^*^provided in a whole foods matrix (cooked beef and ground mealworms, respectively). All data based on phenylalanine. AA, Free amino acids; Casein, Micellar casein; Whey, Whey protein; Milk, Milk protein concentrate; REX, Resistance exercise; END, Endurance exercise; low PRO, Following lower protein diet; high PRO, Following higher protein diet; CHO, Carbohydrate co-ingestion; FAT, Fat co-ingestion; insulin, Insulin infusion; sleep, Overnight sleep; leu, Leucine co-ingestion; immob, Leg immobilization; CWI, Cold-water immersion; HWI, Hot-water immersion.

## 6 Discussion

A variety of methods have been applied to assess postprandial whole-body protein metabolism based on the plasma amino acid kinetics model. The accuracy of these methods differs greatly, based on their capacity to accurately assess exogenous plasma amino acid bioavailability. We introduced a novel exogenous plasma amino acid model based on intrinsically labeled protein-derived reference data that are more accurate than data used in previous estimation approaches.

Extension of amino acid tracer infusion with ingestion of intrinsically labeled protein is the preferred approach to assess exogenous plasma amino acid bioavailability and postprandial whole-body metabolism. When this is not feasible, exogenous plasma amino acid bioavailability can be estimated based on reference data obtained with labeled protein during similar experimental conditions. For example, if an experiment is conducted with the ingestion of 25 g of milk protein in healthy, young adults following resistance exercise with a 6-h post-prandial assessment period, it can be estimated that ~58% of the ingested protein appear will have appeared in the circulation at the end of the assessment period (Table 1). In case of a phenylalanine tracer infusion, the exogenous plasma amino acid bioavailability expressed as percentage of ingested protein would be multiplied with phenylalanine content of milk protein (~6.3 mmol/25 g) and divided by 6-h to calculate the average exogenous rate of plasma amino acid appearance rates. The latter can be used to calculate the average whole-body protein breakdown rate over the full 6-h period. In this example, the estimate would be highly accurate based on appropriate reference data, with only inter-individual differences in protein digestion, amino absorption, and splanchnic extraction not being accounted for. Additional error would be introduced if the experimental conditions differ more from the reference data (e.g., no exact match of dose, population, co-intervention, etc.). It should be carefully considered if there are suitable reference data for extrapolation, to what extent extrapolation errors may impact conclusions, and a brief rationale should be provided in the methods and/or limitations sections. When no suitable exogenous plasma amino acid bioavailability reference data are available (for nearly all non-dairy proteins), the digestibility approach could be applied. Digestibility of many dietary protein source are available (25, 27). However, digestibility scores typically represent the maximal value that would be obtained if the ingested protein is given sufficient time to be absorbed. Therefore, digestibility scores should only be extrapolated to bolus feeding if there is an indication that maximal exogenous plasma amino acid bioavailability can be achieved within the assessed post-prandial period. In the digestibility approach, this could be estimated by a return of plasma amino acid concentrations to basal or control conditions (Figure 2). However, even if the assumption of maximal digestibility has been met, exogenous plasma amino acid availability or whole-body protein breakdown estimated by the digestibility method still differ from those obtained with intrinsically labeled protein as the gold standard (Figure 4B). Therefore, there is an urgent need to obtain more labeled protein-derived exogenous plasma amino acid bioavailability data for various protein sources (e.g., various plant proteins and whole-foods protein sources) This will allow the more routine assessment of postprandial whole-body protein metabolism based on (just) an amino acid tracer infusion to be more accurate when compared to the digestibility or 100% bioavailability methods.

It should be noted that all plasma amino acid kinetics have some limitations. It is assumed that amino acids taken up from the circulation are either incorporated into tissue protein (protein synthesis) or catabolized (amino acid oxidation). However, there can also be a (transient) expansion of tissue-free amino acid pool after bolus feeding. At least in muscle tissue (often referred to as the largest protein pool in the body), such expansion has returned to baseline in <4 h for phenylalanine, but not for the branched-chain amino acids (12). Another limitation is that the metabolic outcomes are assessed based on the kinetics and of single amino acid tracer and extrapolated to all amino acids and/or protein. A final limitation is that the plasma amino acid kinetics model only accounts for fluxes into and out of the circulation. For example, intracellular amino acid (re)cycling may occur. Therefore, actual protein breakdown and protein synthesis rates are likely higher than observed based on plasma amino acid kinetics, although it should not impact protein balance. Another example of a protein flux that is not observed is the loss of endogenous amino acids into the gastrointestinal tract with no reabsorption, which may be far from negligible (52). Therefore, the plasma amino acid kinetics model will need to evolve further to become more complete and accurate. Furthermore, it should be questioned what inferences can be made from the assessment of whole-body protein metabolism. Whole-body protein metabolism reflects the cumulative protein metabolism of all individual organs and tissues. The contribution of an individual organ to whole-body protein metabolism is determined by its individual tissue protein mass and protein metabolic rate. When an increase in whole-body protein synthesis is observed, it cannot be inferred which tissues have contributed to this effect and by what magnitude. Generally, the anabolic response to feeding on a muscle and whole-body level tend to correspond (12, 38, 40). Whether such a relationship exists for other organs remains unclear, as most of these tissues cannot be routinely sampled. Resistance exercise can stimulate muscle protein synthesis rate without a large impact on whole-body protein synthesis rates (42, 47). While muscle tissue represents the largest protein pool, muscle tissue protein synthesis rates are much lower than tissue protein synthesis rates of most organs (11). Therefore, insights on tissue specific and whole-body protein metabolism should be taken into account when evaluating the impact of interventions, populations and/or conditions on protein metabolism.

## 7 Conclusion

The application of the labeled protein model can accurately assess exogenous plasma amino acid bioavailability and, as such, postprandial protein metabolism. However, the cost of producing and applying intrinsically labeled protein limits widespread application. Exogenous plasma amino acid bioavailability can be estimated based on reference data obtained with labeled protein during similar experimental conditions. If no appropriate plasma bioavailability data are available, the digestibility approach can be applied. Application of these models requires an understanding of their underlying assumptions and the consequences when violating them. There is an urgent need for more exogenous plasma amino acid bioavailability data on common dietary protein sources in various experimental contexts to facilitate the accurate assessment and/or estimation of postprandial whole-body protein metabolism.

## Author contributions

JT: Conceptualization, Visualization, Writing – original draft. LL: Conceptualization, Writing – review & editing.
